# Mechanisms of Tanshinone II a inhibits malignant melanoma development through blocking autophagy signal transduction in A375 cell

**DOI:** 10.1186/s12885-017-3329-y

**Published:** 2017-05-22

**Authors:** Xiaojing Li, Zhifeng Li, Xianping Li, Baoguo Liu, Zhijun Liu

**Affiliations:** Department of Dermatology, Affiliated Hospital of Hebei University of Engineering, Handan, Hebei Province 056002 People’s Republic of China

**Keywords:** Tanshinone II A, Malignant melanoma (MM), A375 cell, Autophagy, Cell invasion and migration

## Abstract

**Background:**

Malignant melanoma (MM) is one of the high degree of malignancy and early prone to blood and lymph node metastasis. There is not cured for MM. Tan II A has been reported to reduce cancer cell proliferation. But the mechanism by which Tan II A inhibited melanoma growth are not well characterized. We sought to explore the possible mechanism by which Tan II A regulated cell proliferation through autophagy signaling pathway in A375 cells.

**Methods:**

We tested the effects of Tan II A on melanoma A375, MV3, M14, and other human cell lines including Hacat and HUVEC cells in cell culture model. Cell proliferation was assessed by using methyl thiazol tetrazolium (MTT) assay. Cell migration ability melanoma A375 was monitored by using cell scratch assay. Transwell chamber experimental was performed to assess the effect of Tan II A on A375 melanoma cell invasion ability. The autophagy body was examined by using flow cytometry. The expression of autophagy-associated protein beclin-1 and microtubule-associated protein 1 light chain 3(LC3)-II, as well as phosphatidylinositol 3-kinase(PI3K)、protein kinase B (Akt)、mammalian target of rapamycin (mTOR)、p70S6K1 signaling pathways were detected by using Western blotting. The effects of Tan II A on tumor progression was also examined in melanoma A375 induced tumor in mouse model.

**Results:**

We found that Tan IIA inhibited melanoma A375, MV3, and M14 cell proliferation in dose and time dependent manner. Tan II A reduced CXCL12-induced A375 cell invasive ability and migration in a dose dependent manner. Tan IIA promoted autophagic body production and increased autophagy-associated protein beclin-1 and LC3-II expression in A375 cells. However, Tan IIA reduced the phosphorylation of PI3K, P-AKT, P-mTOR, and P-p7036k1. We also confirmed that Tan II A reduced melanoma A375 induced tumor volume and weight in mouse model.

**Conclusions:**

We concluded that Tan II A reduced A375 cells proliferation by activation of autophagy production, blocked PI3K- Akt – mTOR - p70S6K1 signaling pathway, increased autophagic related gene beclin-1, LC3-II protein expressions and induced autophagocytosis. Tan II A inhibited melanoma A375 induced tumor development in mouse model.

## Background

Malignant melanoma (MM) is one of the high degree of malignancy and early prone to blood and lymph node metastasis [1–3]. Surgery to remove the tumor and chemotherapy are the routine treatments for early-stage melanoma. However, these treatments cannot effectively control the recurrence and distant metastasis. Autophagy (or autophagocytosis) is a type II programmed cell death in respond to the non -invasive persistent internal and external stimulation and stress in eukaryotic cells [4, 5]. Autophagy is a natural process to orderly degrade and recycle cellular components [6]. The host cell exerts its self-clearing of toxic substances such as damaged proteins and organelles through autophagy processes [7]. Autophagy plays an important role in cell growth, development and disease suppression. For example, it has been shown that the occurrence and development is closely related to autophagy and tumor [4]. When the cell’s DNA and protein damaged, the cells maintained its cellular homeostasis through autophagy. If cell autophagy function failed, DNA damage will increase the cell incidence of cancer transformation [4]. It has been reported that the reduction of autophagy-related gene expressions in skin melanoma [8]. However, there was report that autophagy helped to maintain the survival of tumor cells which defected apoptosis ability [9]. In esophageal cancer, radiation therapy was found inducing autophagy in cancer cells, promoting cancer cell proliferation and causing treatment resistance [10]. Other reports suggested that autophage suppressed cancer development in early stage and promoted cancer cell proliferation in later stage [4]. Therefore, autophagy may play dual functions in cancer cell development and progression through apoptosis process [5]. It has become important to understand autophagocytosis functions in clinical treatments seeking to suppress MM invasion and metastasis. Tanshinone IIA (TanIIA) is a fat-soluble Chinese medicine extract which ingredient can inhibit tumor cell growth, induce cell apoptosis and differentiation [11, 12]. In this study, we sought to explore the possible mechanism by which TanIIA affected melanoma cell proliferation, invasion, and migration through autophagy regulated gene expression and its signaling transduction pathways in cell culture models and animal mouse models.

## Methods

### Cell culture

Melanoma cell lines including A375, MV3, M14, and other cell lines including human Hacat (spontaneously transformed aneuploid immortal keratinocyte cell line from adult human skin), human umbilical vein endothelial cells (HUVEC) cells A375 cells were purchased from American Type Culture Collection (ATCC, Manassas, VA 20108, USA) [13–18]. The cells were grown in 10% FBS Dulbecco’s minimum essential medium (DMEM) (GIBCO, Thermo-Fisher, USA)in an incubator with 37 °C, 5% CO2. Tan IIA standards were purchased from Chinese Academy of Inspection Quarantine Test Center.

### MTT cell proliferation assay

Melanoma A375, MV3, M14 cells, and other human cell lines including Hacat or HUVEC cells were seeded in 96-well plates with 5 × 10^3^/100 μL containing with 0.5, 1.0 or 4.0 μg/mL TanIIA for 24, 48, or 72 h culture respectively. The cells with dimethyl sulfoxide (0.1% DMSO, 0.0 μg/mL Tan II A) was used as negative control. Each treatment included six duplicate wells. To measure cell viability, 20 μl of MTT (5 μg/μL) (ATCC, Manassas, VA 20108, USA) was added to each well and incubated for 4 h at 37 °C in culture hood. The media was carefully removed and 150 μl MTT solvent was added to each well. The plate was covered with tinfoil and agitated on orbital shaker for 15 min. The absorbance (OD) at 490 nm (A) value was measured by micro-plate reader to determine the cells proliferation activity. The above experiment was repeated three times. The MTT reading level in negative control cell was used as 0% cell inhibition. The MTT reduction amount in Tan II A treated cells compared to negative control was normalized with negative control (0.1% DMSO, 0.0 μg/mL Tan II A) MTT level to calculate the inhibition percentage.

### Transwell chamber experimental settings to assess the effect of TanIIA on A375 melanoma cell invasion ability

Transwell chamber was purchased from Corning Corporation, USA. These Transwell chamber was coated with polycarbonate membrane matrigel gel that made artificial basement membrane. TanIIA (1, 2, or 4 μg/mL) treated A375 cells for 48 h were trypsin digested, counted, washed, and adjusted to 0.6 × 10^6^ cell/ml in DMEM without FBS. The 24-well transwell top chamber was 1st incubated with 500 μl of DMEM without FBS for 2 h. The appropriate top chamber DMEM was then replaced with 300 μl of TanIIA (1, 2, or 4 μg/mL) treated A375 cells without FBS DMEM (0.6 × 10^6^ cell/ml). The 500 μl of DMEM 10% FBS with 10 ng/ml of CXCL12 was added to the bottom chamber. The cells were incubated in the transwell for 24 h. The bottom chamber of transwell membrane was then stained with CeU Counting Kit-8 (CCK-8) (Dojindo Research Laboratories, Japan) for 20 min. The cells were washed with PBS to remove excess dye, dried, and counted the number of cells passed through the membrane under a microscope (200 X amplification). All experiments were repeated three times.

#### Cell scratch assay to test TanIIA effect on A375 cells migration

A375 cells were treated with TanIIA (1, 2, or 4 μg/mL) for 48 h in 12 well plate (6 × 10^4^ cell/1 ml. The sterilized P200 pipette tip was used to scratch on the monolayer cells to create a straight line. We took extra attention to create scratches of approximately similar size in the assessed cells and control cells to minimize any possible variation caused by the difference in the width of the scratches. The cells were washed with 1 ml PBS for three times and cultured with 2 ml 10% FBS DMEM for 48 h. The cells and scratches images were taken and measured by a phase-contrast microscope (200 X amplification) at 0, 24, and 48 h after scratch. To obtain the same field during the image acquisition, we created markers as reference points close to the scratch. The reference points were made by etching the dish lightly with a razor blade on the outer bottom of the dish or with an ultrafine tip marker. After the reference points were made, we placed the dish under a phase-contrast microscope, and left the reference mark outside the capture image field but within the eye-piece field of view. For each image, we measured the interval distances in micrometer (μM) between one sides of scratch to the other. All experiments were repeated three times.

### Flow cytometry to quantitative detect the effect of TanIIA on autophagic body production in A375 cells

A375 cells were treated with TanIIA (1, 2, or 4 μg/mL) for 48 h in 6 well plate (5 × 10^5^ cell/2 ml at 37 °C and 5% CO2. The cells with 0.1% DMSO (0 μg/ml Tan II A) was used as negative control. The treated cells were washed with PBS, trypsin digested, and harvested to the 5 ml Falcon round-bottom polystyrene tubes. The collected cells were stained with acridine orange (1 μg/1 mL) at room temperature in the dark for 20 min. The dye was discarded after centrifugation with 3 ml PBS washing buffer for three times. The cells were resuspended in 1 ml PBS for flow cytometry analysis. FL1 and FL3 channel is for detection of green and red fluorescence respectively. FL3 red fluorescence reflects the strength of autophagy body number.

### Western blot to assess the effect of tan II a on autophagy related protein expression

A375 cells were treated with TanII A (1, 2, or 4 μg/mL) for 48 h in 6 well plate (5 × 10^5^ cell/2 ml) at 37 °C and 5% CO2. The cells with 0.1% DMSO was used as negative control. At the time of harvest, cells were washed with PBS, and whole cell protein samples were extracted with radioimmunoprecipitation assay buffer (0.5% Nonidet p-40, 10 mM Tris, pH 7.4, 150 mM NaCl, 1% SDS) with a protease inhibitor cocktail (Sigma Life Science and Biochemicals, St. Louis, MO). Protein (70 μg/well) was separated by SDS-PAGE and transferred to PVDF membranes. The primary antibodies used for the Western blots included mouse anti-Beclin-1, mouse anti-microscopic light chain I protein 3 (the microtubule-associated protein light chain3, LC3) -II, and mouse anti-β-actin (Santa Cruz Biotechnology, TX, USA); mouse anti-phosphatidylinositol 3-kinase (PI3K), mouse anti- phosphorylated (p) - protein kinase B (P-Akt), p- mammalian target of rapamycin (P-mTOR), p-p70S6K1 (Cell Signaling Co, MA, USA). The secondary antibodies included HRP-conjugated ECL sheep anti-mouse IgG (GE Amersham Biosciences, USA). The ECL Western Blotting Detection Kit (GE Amersham Biosciences, USA) was used to detect Chemiluminescent signals in the X-ray film. The GAPDH was used as internal protein level control. Each experiment was repeated three times.

### Construction of mouse animal model to assess the effect of tan II a on A375 melanoma induced tumor development

All animal experiments were approved by the Affiliated Hospital of Hebei University Animal Policy and Welfare Committee and complied with the NIH guideline (Guide for the care and use of laboratory animals). Wild type (WT) BA LB/e nude mice were obtained from Shanghai SLAC Laboratory Animal CO. LTD (Shanghai, China). We tested the effects of Tan II A on the transplanted skin melanoma tumor progression in mouse. We harvested cell culture A375 cells. Each of 1X10^6^/100 μl A375 cell suspension was subcutaneously inoculated to each mouse right armpit to induce the tumor. We started to measure tumor size at Day 14 post tumor transplantation. The tumor volume (TV) was calculated by this formula: TV = (1/2)*a*b2. (a and b was tumor length and width respectively). At 14 days post transplantation when the tumor volume was about 50–100 mm^3^, the mice were randomly assigned to four groups with 6 mice in each group. We inoculated 100 μl of PBS (Untreated Control), Paclitaxel (8 μg/g) once every 2 days, Tan II (A) (25 μg/g) or Tan II (A) (50 μg/g) once every day to the appropriate mouse for 28 days. The mice were sacrificed at day 28 post treatments. The tumors were harvested, weighted, and recorded.

### Statistical analysis

SPSS17.0 statistical software was used for ANOVA and Dunnett test data analysis. Data are expressed as mean ± SD of at least three sample replicates, unless stated otherwise. Data analysis was performed using a 2-tailed Student’s t-test. *P < 0.05, ** *P* < 0.01, and ***P < 0.001 for comparison of indicated treatments.

## Results

### TanIIA inhibited melanoma A375, MV3, M14 cells and other human cell Hacat, HUVEC cell proliferation in dose and time dependent manner

**Fig. 1:**
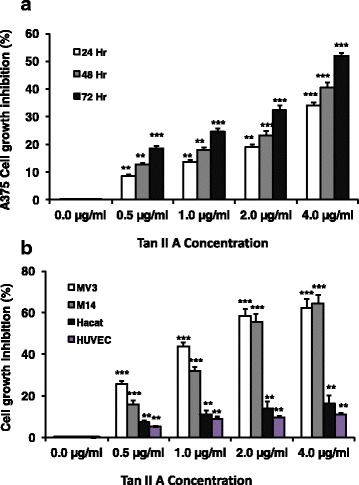
Tan II A inhibited A375, MV3, M14, Hacat, and HUVEC cells proliferation in a dose and time dependent manner. A375 cells were treated with Tan IIA (0.5, 1, 2, or 4 μg/mL) for 24, 48, or 72 h respectively. MV3, M14, Hacat (spontaneously transformed aneuploid immortal keratinocyte cell line from adult human skin), and human umbilical vein endothelial cells (HUVEC) cells were treated with Tan IIA (0.5, 1, 2, or 4 μg/mL) for 72 h respectively. DMEM 10% FBS with DMSO (0.1%) (0.0 μg/mL Tan II A) was used as the negative control. The MTT reading level in the negative control cell was used as 0% cell inhibition. The MTT reduction amount in Tan II A treated cells compared to the negative control was normalized with the negative control (0.1% DMSO) MTT level to calculate the inhibition percentage. *P < 0.05, ** *P* < 0.01, and ***P < 0.001 for comparison of indicated treatments to the negative control at 24, 48, or 72 h respectively. *n* = 6 for treatment. **a**. Tan IIA treatment significantly increased the inhibition of A375 cells proliferation at 24, 48, and 72 h in 0.5, 1, 2, or 4 μg/mL Tan II A compared to the respectively negative controls. **b**. Tan IIA treatment significantly enhanced the inhibition of MV3, M14, Hacat, and HUVEC cells proliferation at 72 h in 0.5, 1, 2, or 4 μg/mL Tan II A compared to the respectively negative controls

To assess the effects of TanIIA on melanoma cell proliferation, we treated A375 cells with TanIIA at (0.5, 1.0, 2.0, or 4.0 μg/mL) for 24, 48, or 72 h respectively. DMEM 10% FBS with 0.1% DMSO (0.0 μg/mL Tan II A) was used as negative control. We found that TanIIA treatment significantly inhibited A375 cells MTT levels at 24, 48, and 72 h in 0.5, 1, 2, or 4 μg/mL Tan II A respectively compared to negative control (Fig. 1). Tan II A had stronger inhibition on A375 cell proliferation with higher concentration (from 4, 2, 1 to 0.5 μg/mL) at 24, 48, and 72 h respectively compared to the negative control. Longer culture time (from 72, 48 h to 24 h) with Tan II A showed stronger inhibition on A375 cells proliferations in each tested concentration of Tan II A compared to the earlier time (Fig. 1). These findings indicated that TanIIA inhibitions on A375 cells growth were in dose and time dependent manner (Fig. 1). To further determine the effects of Tan II A on other melanoma or other normal cell lines proliferation, we treated melanoma MV3, M14 cells and normal cell lines Hacat, HUVEC cells with TanIIA at (0.5, 1.0, 2.0, or 4.0 μg/mL) for 72 h. We found that Tan II A treatments significantly reduced the cell growth in all four cell lines compared to the negative control (0.0 μg/mL Tan II A) (Fig. 1b). However, TanIIA exerted larger inhibition on cell growth in both melanoma MV3 and M14 cells compared to the Hacat or HUVEC cells in each tested Tan II A concentration (F = 820.218,*P* < 0.001). These findings further confirmed the Tan II A inhibitory effects on melanoma growth.

### TanIIA reduced CXCL12-induced A375 cell invasive ability in a dose dependent manner

**Fig. 2:**
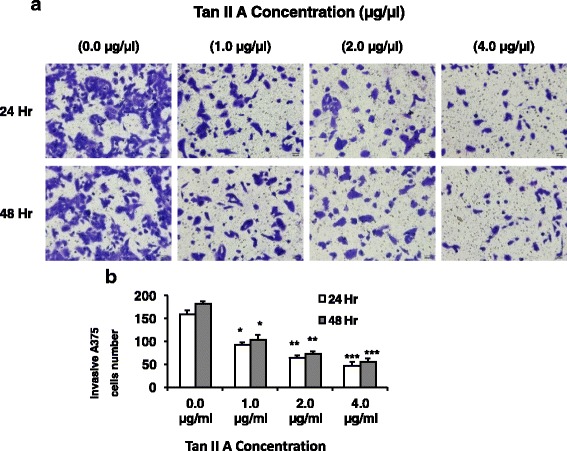
TanIIA reduced CXCL12-induced A375 cell invasive ability in a dose dependent manner. To test the effects of Tan II A on melanoma cell invasion, A375 cells were incubated with TanIIA at 0, 1, 2, or 4 μg/mL for 24 or 48 h. The tested cells were trypsin digested, counted, washed, and adjusted to 0.6 × 10^6^ cell/ml. The treated cells were placed in top chamber of transwell with 10 ng/ml chemokine CXCL12 for 24 h to induce the cell invasion. The A375 cells traveled to the bottom chamber were then stained with CeU Counting Kit-8 (CCK-8). The cell image and number were recorded and counted. **a**. The images of invasive A375 cells in the bottom chamber under a microscope (200 X amplification). **b**. Tan II A significantly decreased A375 cells invasive ability in a dose dependent manner. Each treatment was repeated three times (*n* = 3). *P < 0.05, ** *P* < 0.01, and ***P < 0.001 for comparison of indicated treatments to the negative control (0 μg/mL) at 24 or 48 h respectively

To measure the effects of Tan II A on melanoma cell invasion, A375 cells were incubated with TanIIA (0, 1, 2, or 4 μg/mL) for 24 or 48 h. The treated cells were placed in top chamber of transwell with 10 ng/ml chemokine CXCL12 for 24 h to induce the cell invasion. The A375 cells traveled to the bottom chamber were observed by microscopy (Fig. 2a). We found that 1, 2, or 4 μg/mL Tan II A significantly decreased A375 cell invasion numbers compared to negative control (0 μg/mL) at 24 or 48 h (Fig. 2b). Higher concentration (from 4, 2, to 1 μg/mL) of Tan II A had stronger inhibition on A375 cell invasion ability compared to the negative control. These observations suggested that TanIIA inhibitions on A375 cells invasion were in dose dependent manner (Fig. 2).

### TanIIA decreased CXCL12-induced A375 cell migration

**Fig. 3:**
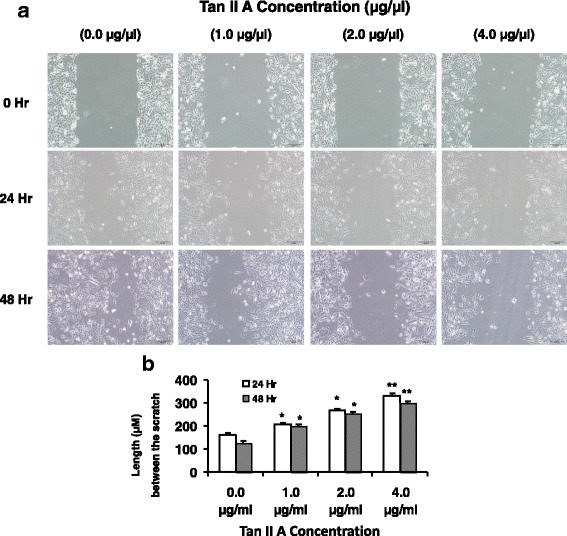
TanIIA decreased CXCL12-induced A375 cell migration. To test the effects of Tan II A on melanoma cell migration, A375 cells were incubated with TanIIA (0, 1, 2, or 4 μg/mL) for 48 h in 12-well plate. The sterilized P200 pipette tip was used to create a straight line scratch on the monolayer cells. The cells in each well were treated with 10 ng/ml chemokine CXCL12 for 48 h. We monitored the cells migration to the scratch area at 0, 24 and 48 h. For each image, the interval distance in micrometer (μM) between one sides of scratch to the other was measured. **a**. The images of A375 cells in 12- well with one scratch line under a microscope at 0, 24, or 48 h post scratch (200 X amplification). **b**. Tan II A significantly increased scratch clear length in a dose dependent manner. Each treatment was repeated three times (*n* = 3). *P < 0.05, ** *P* < 0.01, and ***P < 0.001 for comparison of indicated treatments to the negative control (0 μg/mL) at 0, 24 or 48 h post scratch respectively

To test the effects of Tan II A on melanoma cell migration, A375 cells were incubated with TanIIA (0, 1, 2, or 4 μg/mL) for 48 h in 12-well plate. We used the sterilized P200 pipette tip to scratch on the monolayer cells to create a straight line. The cells in each well were treated with 10 ng/ml chemokine CXCL12 for 48 h. We monitored the cells migration to the scratch area at 0, 24 and 48 h. We found that A375 cells migrated to the scratch area in the wells without Tan II A treatment. However, there are more clear scratch fields without A375 cells in wells with higher concentration of Tan II A (2, 4 μg/mL) compared to the negative control (Fig. 3a). We found that 1, 2, or 4 μg/mL Tan II A significantly increased the scratch clear length in micrometer (μM) compared to the negative control (0 μg/mL) at 24 or 48 h (Fig. 3b). Higher concentration (from 4, 2, to 1 μg/mL) of Tan II A had longer clear field compared to the negative control. These findings indicated that TanIIA inhibitions on CXCL12-induced A375 cells migration were in dose dependent manner (Fig. 3).

### TanIIA promoted autophagic body production in A375 cells

**Fig. 4:**
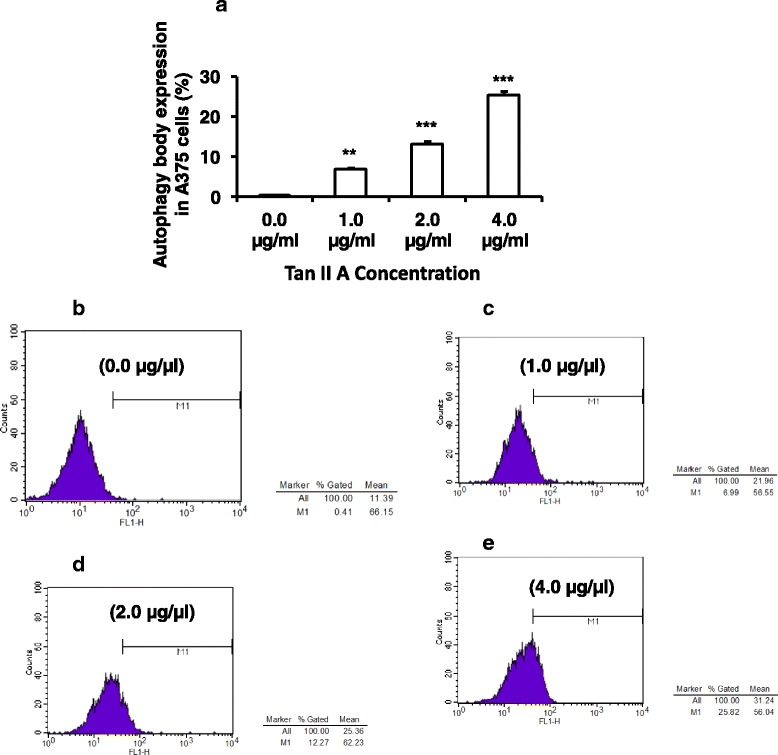
TanII A promoted autophagic body production in A375 cells. To assess the effects of TanIIA on autophagic body production in A375 cells, A375 cells were incubated with TanIIA at (0, 1.0, 2.0, or 4.0 μg/mL) respectively for 48 h. The cells were stained with acridine orange for flow cytometry analysis to monitor autophagic body production. **a**. TanIIA enhanced autophagic body production on A375 in a dose dependent manner. Each treatment was repeated three times (*n* = 3). *P < 0.05, ** *P* < 0.01, and ***P < 0.001 for comparison of indicated Tan II A treatments to the negative control (0 μg/mL Tan II A) respectively. **b**–**e**. The representative flow cytometry histograms of autophagic body staining on A375 cells treated with 0, 1, 2, or 4 μg/mL Tan II A respectively

Autophagy has been shown associated with cancer cell proliferation. To assess the effects of TanIIA on autophagic body production in A375 cells, we treated A375 cells with TanIIA at (0, 1.0, 2.0, or 4.0 μg/mL) respectively for 48 h. The cells were stained with acridine orange for flow cytometry analysis to monitor autophagic body production. We found that TanIIA treatment significantly increased autophagic body production compared to the negative control in A375 cells (Fig. 4). Higher concentration (from 4, 2, to 1 μg/mL) of Tan II A had stronger stimulation on autophagic body production in A375 cell compared to the negative control. These observations indicated that TanIIA promoted autophagic body production on A375 in dose dependent manner (Fig. 4).

### TanIIA regulated autophagic related genes and signaling pathway in A375 cells

**Fig. 5:**
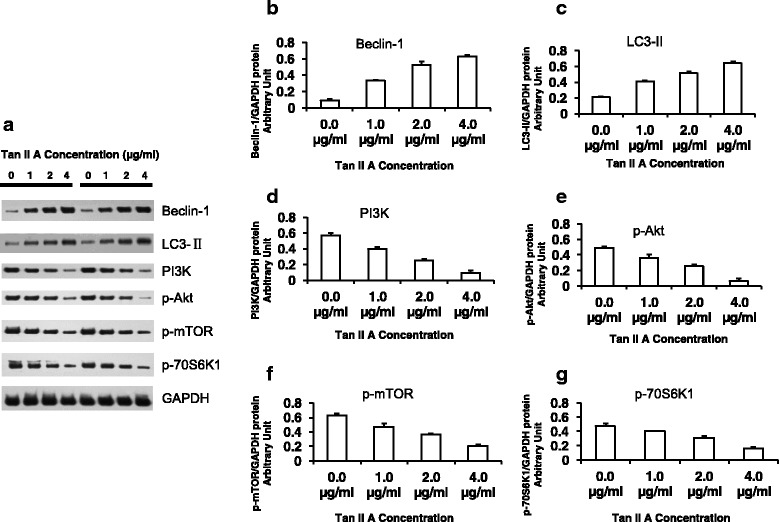
TanIIA regulated autophagic related genes protein expression and signaling pathway in A375 cells. A375 cells were treated with TanII A (0, 1, 2, or 4 μg/mL) for 48 h in 6 well plate (5 × 10^5^ cell/2 ml). The cells protein lysates were harvested for Western blot. The primary antibodies used for the Western blots included mouse anti-Beclin-1, mouse anti-LC3 -II, mouse anti-PI3K, mouse anti- phosphorylated (p) - protein kinase B (P-Akt), p- mammalian target of rapamycin (P-mTOR), p-p70S6K1. The GAPDH was used as internal protein level control. **a**. The Western blot images for anti-Beclin-1, LC3 -II, PI3K, P-Akt, P-mTOR, p-p70S6K1, and GAPDH in A375 cells treated with Tan II A. Each concentration showed two duplicate treatments. **b**. Tan II A increased Beclin-1 protein expression in a dose dependent manner. **c**. Tan II A enhanced LC3-II protein expression in a dose dependent manner. **d**. Tan II A decreased PI3K protein expression in a dose dependent manner. **e**. Tan II A reduced P-Akt protein phosphorylation in a dose dependent manner. **f**. Tan II A inhibited P-mTOR protein phosphorylation in a dose dependent manner. **g**. Tan II A decreased P-p70S6K1 protein phosphorylation in a dose dependent manner. Each bar was the average of three replicates (*n* = 3). *P < 0.05, ** *P* < 0.01, and ***P < 0.001 for comparison of indicated Tan II A treatments to the negative control (0 μg/mL Tan II A) respectively

To further analyze the effects of Tan II A on regulation of autophagic related genes expression and pathway signaling, we performed Western blot to assess the related protein expression. We found that TanII A treatment significantly increased Beclin-1 and LC3-II (the microtubule-associated protein light chain3-II) protein expression respectively. Higher concentration (from 4, 2, to 1 μg/mL Tan II A) exerted stronger Beclin-1 and LC3-II protein expression enhancement compared to the negative control (Fig. 5a, b-c). These findings indicated that Tan II A increased autophagic related gene protein expression in a dose dependent manner. In contrast, we found that TanII A treatment significantly reduced the protein phosphorylation of phosphatidylinositol 3-kinase (PI3K), phosphorylated (p) - protein kinase B (P-Akt), p- mammalian target of rapamycin (P-mTOR), and p-p70S6K1 respectively. Higher concentration (from 4, 2, to 1 μg/mL Tan II A) had more reduction on related protein phosphorylation compared to the negative control (Fig. 5a, d-g). These observations suggested that Tan II A inhibited autophagic related gene signaling pathway in a dose dependent manner. These results suggested that TanIIA regulated autophagic production, Beclin-1, LC3-II and subsequently PI3K-Akt-mTOR-p70S6K1 signaling pathway in A375 cells.

### Tan II a inhibited A375 melanoma induced tumor development in mouse

**Fig. 6:**
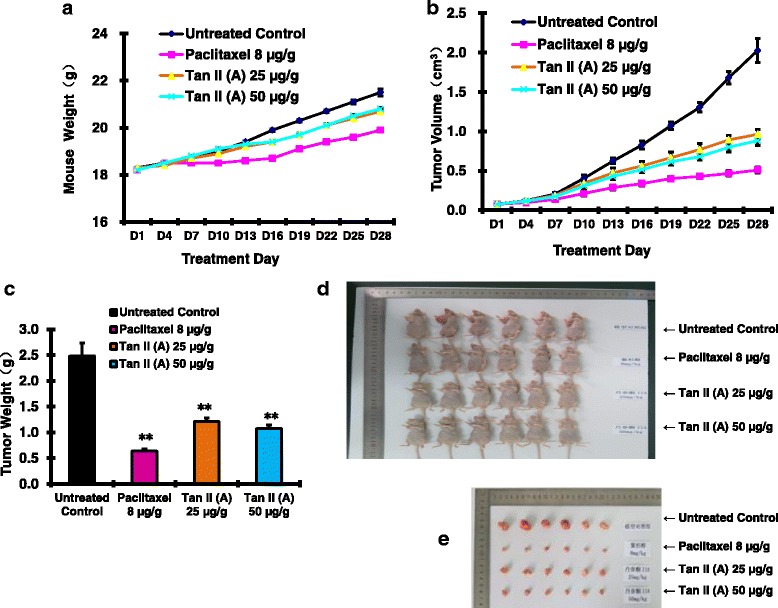
TanIIA inhibited the melanoma A375 cell induced tumor development in the mouse model. The mice were inoculated with the melanoma A375 cells to induce tumor as described in the Methods. At 14 days post transplantation when the tumor volume was about 50–100 mm^3^, the mice were randomly assigned to four groups with 6 mice in each group. The selected mice were inoculated with 100 μ of PBS (Untreated Control), Paclitaxel (8 μg/g), Tan II (A) (25 μg/g), or Tan II (A) (50 μg/g) respectively. The mice were sacrificed at day 28 post treatments. The tumors were harvested, weighted, and recorded. **a**. TanIIA and Paclitaxel treatments modestly reduced mouse weight. **b**. TanIIA and Paclitaxel treatments significantly inhibited tumor volume. **c**. TanIIA and Paclitaxel treatments significantly reduced tumor weight at day 28 post treatments. **d**. The display image of of harvested mice from Untreated Control, Paclitaxel (8 μg/g), Tan II (A) (25 μg/g), or Tan II (A) (50 μg/g) treatments respectively. **e**. The visualize images of mice tumor. TanIIA and Paclitaxel treatments significantly decreased tumor size at day 28 post treatments

We found that Paclitaxel (8 μg/g), Tan II (A) (25 μg/g), or Tan II (A) (50 μg/g) treatments modestly reduced the mouse weight compared to the untreated control (Fig. 6a). As expected, Paclitaxel (8 μg/g) treatments significantly inhibited A375 cell transplanted skin melanoma tumor volume (Fig. 6b) compared to untreated control. We also found that both 25 μg/g and 50 μg/g Tan II (A) treatments significantly reduced A375 cell transplanted skin melanoma tumor volume compared to the untreated control (Fig. 6b). Moreover, at 28 day post the treatment, Paclitaxel (8 μg/g), Tan II (A) (25 μg/g), or Tan II (A) (50 μg/g) treatments significantly reduced A375 cell transplanted skin melanoma tumor weight compared to the untreated control (Fig. 6c). We observed the significantly smaller tumor sizes in Paclitaxel (8 μg/g), Tan II (A) (25 μg/g), or Tan II (A) (50 μg/g) treatments compared to the untreated control (Fig. 6b-e). These findings confirmed the Tan II (A) inhibitory effects on A375 melanoma induced tumor development in mouse.

## Discussion

There is not magic cured for malignant melanoma (MM) [1–3]. Surgery and chemotherapy are the routine therapies to suppress the MM progression. But these treatments cannot effectively control the recurrence and distant metastasis. There are several MM suppressive drugs or compounds which associated with autophagy induction. For example, roselle (*Hibiscus sabdariffa*) leaf extract increased the expressions of autophagy-related proteins including autophagy-related gene 5 (ATG5), beclin1, light chain 3-II (LC3-II), and induced autophagic cell death in A375 cells [19]. Curcumin (the yellow spice derived from the rhizome of *Curcuma longa*) effectively inhibited the proliferation and invasion through autophagy induction in A375 and C8161 cells [20]. Imiquimod increased autophagy and apoptosis to induce cell death in a dose and time dependent manner in basal cell carcinoma (BCC) cells [21]. Tan II A has been shown to inhibit esophageal, colon, and other cancers proliferation through the induction of cell apoptosis and differentiation [22, 23]. Tan II A induced intracellular generation of reactive oxygen species (ROS) to regulate autophagy and apoptosis in lung cancer cell [11]. Tan II A activated AMPK, ERK and suppressed mTOR, p70S6 K to induce autophagy and apoptosis in leukemic KBM-5 cells [12]. Light chain micro-protein 3 (LC3) is autophagy universal marker which included two subtypes LC3-I and LC3-II. LC3-I is a soluble cytoplasmic protein expressed in normal condition. When cell initiates autophagocytosis, LC3-I interacts with autophagic protein PE by ubiquitin-like modification process to transform to LC3-II membrane protein [24]. LC3-II expression reflects to the mature stages of autophagy. LC3-II levels associate with autophagic bodies in cell and is a convention marker for autophagy activity [24]. Beclin-1 plays an important role in promoting the formation of autophagic vesicles during the cell autophagocytosis process in melanoma [8]. We therefore monitored both LC-II and beclin-1 protein expression to assess their association with autophagocytosis. In this report, we found that Tan II A inhibited melanoma A375 cells proliferation in a dose and time dependent manner. Higher concentration of Tan II A (from 4, 2, 1 to 0.5 μg/mL) and longer culture time (from 72, 48 h to 24 h) showed stronger inhibition on A375 cells proliferations compared to the negative control. Tan II A also significantly inhibited other melanoma MV3 and M14 cell and modestly reduced other human cell line including Hacat, HUVEC growth. We observed that Tan IIA reduced A375 cells invasion in a dose dependent manner. Tan II A also decreased the CXCL12-induced A375 cell migration [25]. Moreover, TanII A promoted autophagic body production on A375 in a dose dependent manner. Tan II A up-regulated the autophagocytosis related genes including Beclin-1 and LC3-II protein expressions in a dose dependent manner. However, TanII A inhibited the phosphorylation of phosphatidylinositol 3-kinase (PI3K), phosphorylated (p) - protein kinase B (P-Akt), p- mammalian target of rapamycin (p-mTOR), and p-p70S6K1 in A375 cells. The PI3K- AKT– mTOR- p70S6K1 signaling pathway plays important physiological roles in both normal and tumor cell growth and proliferation [26, 27]. The activation of this signaling pathway may promote cell proliferation, tumor invasion and metastasis through blocking apoptosis [26, 27]. Our Studies demonstrated that Tan II A inhibited the PI3K- Akt – mTOR - p70S6K1 signal transduction pathway and activated autophagy production. Tan II A was also demonstrated to inhibit the melanoma A375 cell induced tumor progression in the mouse model.

## Conclusion

In summary, based on our experimental data and observation, we found that Tan II A reduced A375 cells proliferation by activation of autophagy pathway. We therefore proposed a model in which Tan II A inhibited A375 cells proliferation by blocked PI3K- Akt – mTOR - p70S6K1 signaling pathway, increased autophagic related gene beclin-1, LC3-II protein expressions and induced autophagocytosis. We speculated that PI3K-Akt-mTOR-p70S6K1 pathway might be a novel target for cancer treatment research. Our study provided new data and more evidence of Tan II A inhibited cancer cell proliferation through blocking the PI3K- Akt – mTOR - p70S6K1 signaling pathway and subsequently activation of autophagocytosis pathway in A375 cells. Tan II A decreased the melanoma A375 cell induced tumor development in the mouse model.

## Abbreviations

Hacat: Spontaneously transformed aneuploid immortal keratinocyte cell line from adult human skin)
HUVEC: Human umbilical vein endothelial cells
MM: Malignant melanoma
MTT: Methyl thiazol tetrazolium assay
p: phosphorylation of
P-Akt: Phosphorylated (p) - protein kinase B
PI3K: Phosphatidylinositol 3-kinase
P-mTOR: P- mammalian target of rapamycin (P-mTOR)
Tan II A: Tanshinone II A
